# Lidocaine Injection Provides Superior Pain Relief Compared to 10-Minute BLT Topical Numbing in Women Undergoing Cannula-Based Aesthetic Procedures

**DOI:** 10.1093/asjof/ojag005

**Published:** 2026-01-20

**Authors:** Chin-King Looi, Yi Shan Phoon, Lina Pei Shi Yow, Chun-Wai Mai, Tuck Wah Siew

## Abstract

Despite the growing popularity of aesthetic procedures, procedural pain remains a major concern among patients. Topical numbing cream and local anesthetic injection are widely used to manage discomfort and reduce pain; however, their relative effectiveness in cannula-based facial treatments remains a subject of ongoing debate. The aim of the study was to compare the anesthetic efficacy of BLT cream (benzocaine 20%, lidocaine 10%, and tetracaine 4%) and 1% lidocaine local injection in patients undergoing cannula-based facial aesthetic treatments. Retrospectively, 29 females underwent 2 separate treatment sessions, each using a different anesthetic method. Pain intensity was assessed using a self-reported numerical pain rating scale, and patients were asked to indicate their preferred method upon completion of the second treatment session. Mean pain scores were compared using paired samples *t*-test, and patient preference was analyzed using frequency and percentage distributions. A *P*-value of <.05 was considered statistically significant. Of the 29 patients enrolled, 28 patients were included in the final analysis. The mean pain score with BLT cream was 5.07 ± 0.40 (95% CI, 4.24-5.90), compared with 3.04 ± 0.32 (95% CI, 2.38-3.69) with 1% lidocaine injection. The difference was statistically significant (*P* = .0001), with a mean difference of 2.03. Most patients reported a preference for 1% lidocaine injection over topical cream for future treatments. In this preliminary study, local injection of 1% lidocaine provides significantly better pain control than BLT cream during cannula-based facial treatments, with higher patient preference. Injectable anesthesia may be a more effective option for enhancing patient comfort in aesthetic practice.

**Level of Evidence:** 3 (Therapeutic) 
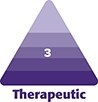

Facial skin quality plays a crucial role in an individual's overall appearance, and poor skin condition can negatively impact self-esteem and quality of life.^1^ In recent decades, the global aesthetic market has experienced exponential growth, driven by increasing demand for facial rejuvenation across diverse age groups, from maintaining a youthful appearance to enhancing facial contours and minimizing scars.^2^ According to Grand View Research, the global aesthetic market is projected to reach USD 143.4 billion by 2030, up from USD 82.5 billion in 2023, growing at a compound annual growth rate of 8.3% from 2024 to 2030.^3^ This rapid growth reflects not only increasing societal acceptance of aesthetic procedures but also significant advancements in aesthetic technologies and treatment modalities.

Facial rejuvenation can be achieved through either invasive or noninvasive modalities. Invasive methods typically involve aesthetic plastic surgery, which enhances facial appearance by repositioning soft tissues to restore youthful contours. Common procedures include eyelid surgery, brow lifts, facelifts, and facial implants.^4^ In contrast, noninvasive or minimally invasive procedures have gained increasing popularity because of their shorter recovery times, lower risk, and more natural-looking results.^5^ These approaches usually involve the administration of biocompatible substances into the subcutaneous or dermal layers, such as botulinum toxin, dermal fillers, synthetic fillers, and platelet-rich plasma, or the use of technologies such as thread lifts and radiofrequency waves.^6,7^

Although these approaches have been proven effective and generally well tolerated, aesthetic procedures are often associated with pain and discomfort, significantly influencing patient decision, and willingness to undergo repeated treatments.^8^ Fear of needles, injections, or pain has been reported as the primary barrier to aesthetic treatments in up to 65% of patients.^9^ Therefore, effective pain management is essential not only to improve patient comfort, satisfaction, and treatment adherence, but also to ultimately improve overall outcomes.

A variety of anesthetic techniques have been explored to alleviate injection-related discomfort and pain, including topical anesthetic creams, vibration and cooling devices, and injectable local anesthetics.^10-14^ These agents function primarily by blocking voltage-gated sodium channels, temporarily blocking nerve impulse transmission, and preventing pain perception.^15,16^ Although topical anesthetics, such as the eutectic mixture of local anesthetics (EMLA) or compounded numbing creams, can reduce discomfort, they often exhibit delayed onset and may not provide adequate pain relief for all patients.^10,17,18^ In contrast, injectable local anesthetics provide more consistent and effective pain control, typically lasting from 30 min to over 12 h.^15^

Given these differences in efficacy, the optimal anesthetic approach in aesthetic practice remains a subject of debate. Therefore, this retrospective study aimed to compare the anesthetic effect and patient preferences between BLT cream (benzocaine 20%, lidocaine 10%, and tetracaine 4%) without any local anesthetic infiltration and 1% lidocaine injection at the injection site during cannula-based facial treatments.

## METHODS

### Study Design, Ethical Consideration, and Recruitment

This retrospective study included all 29 female patients who underwent facial aesthetic procedures at our clinic over an 11-month period (July 2024 to May 2025). The study was conducted in accordance with the Declaration of Helsinki.^19^ Formal Research Ethics Board (REB) review was not required, because all clinical data used in this study were fully anonymized, and the research did not involve any procedures performed specifically for research or cause harm to participants.^20,21^ All previously treated patients were contacted by treating physicians independently from the research team and were fully informed of the research purpose, methods, funding sources, potential conflicts of interest, expected benefits, and risks, as well as their right to refuse participation or withdraw consent at any time without retaliation. Written informed consent was obtained from all participants before enrollment, and patient confidentiality was maintained throughout data collection and analysis. Patients were recruited based on predefined inclusion and exclusion criteria to ensure a representative and appropriate study population. The physicians anonymized the identity of all patients’ data before sending the data to the research team to conduct the analyses.

The inclusion criteria for the study were female patients aged 18 years or older, those undergoing the same facial aesthetic procedures during the study period, and individuals with no history of hypersensitivity to topical anesthetics. The exclusion criteria included male patients, pregnant or lactating women, patients unwilling to provide informed consent, and those with incomplete or missing follow-up data. Patient data, including demographic information, medical history, procedural details, and follow-up outcomes, were retrieved from clinical records. Only patients who completed the planned follow-up were included in the analysis.

### Intervention

All procedures were performed using a 25G, 50 mm cannula (Japan Bio Products Co. Ltd, Tokyo, Japan) introduced through a 23G needle and included injections of skin boosters, dermal fillers, and polynucleotides. Each patient received 2 different anesthetic methods in separate treatment sessions: (1) BLT cream without local anesthetic infiltration during the first session and (2) subcutaneous injection of 1% lidocaine (mixed separately but not included as part of the product) during the second session, typically scheduled 1 to 3 months after the first. The cream was applied by a trained therapist while the lidocaine injection was performed by the doctor who performed the procedure. For the topical anesthetic session, ∼3 g of the BLT cream were uniformly applied to the treatment area and covered with an occlusive cling film dressing for 10 min before the indicated procedure. For lidocaine injection, 0.1 mL of 1% lidocaine was slowly injected subcutaneously at each treatment point using a 30G needle inserted to a depth of 2 to 3 mm, 3 to 5 injection points per side, resulting in a total of 0.3 to 0.5 mL per side.

### Anesthetic Effectiveness and Evaluation

The effectiveness of the 2 anesthetic methods was evaluated using the numerical pain rating scale (NPRS), a validated tool ranging from 0 (no pain) to 10 (worst pain imaginable), as shown in Table 1.^22,23^ After completing both treatment sessions, each patient was asked to rate their pain intensity using the NPRS. In addition to pain assessment, patients were asked to indicate their preferred anesthetic method, based on their personal experience with both techniques. To evaluate the tolerability and safety of each method, the attending clinician qualitatively recorded any local side effects, including erythema, discomfort, and bruising, immediately after the procedure. This approach enabled a combined assessment of pain intensity, patient preference, and treatment-related side effects, providing a subjective comparison of comfort, tolerability, and overall satisfaction associated with each anesthetic technique.

**Table 1.** Classification of Pain Intensity Using the Numerical Pain Rating Scale

Pain score range	Pain intensity	Description
0	None	No discomfort or pain
1-3	Mild	Pain is noticeable but tolerable
4-6	Moderate	Pain is distressing and may interfere with concentration
7-10	Severe	Pain is intense, overwhelming, and significantly interfere with concentration

### Statistical Analysis

All statistical analyses were performed using GraphPad (Boston, MA) Prism (version 9.3.1). Pain scores were recorded and summarized using descriptive statistics. The Shapiro–Wilk test was used to assess the normality of the pain score differences. As the data were not normally distributed, the Wilcoxon matched-pairs signed rank test was used to compare pain scores between the 2 anesthetic methods. Pain scores are presented as mean ± standard error of mean, along with 95% CIs where appropriate. Patient preference data were analyzed using frequency and percentage distributions. A *P*-value of <.05 was considered statistically significant.

## RESULTS

A total of 29 patients were assessed for eligibility, and 1 patient did not report a pain score following the 1% lidocaine injection and was therefore excluded from the analysis. The mean age of participants was 46.29 years (range, 30-71 years). The procedures performed following anesthesia included polynucleotides (*n* = 22, 78.6%), skin booster (*n* = 2, 7.1%), and filler (*n* = 4, 14.3%).

**Figure 1.**
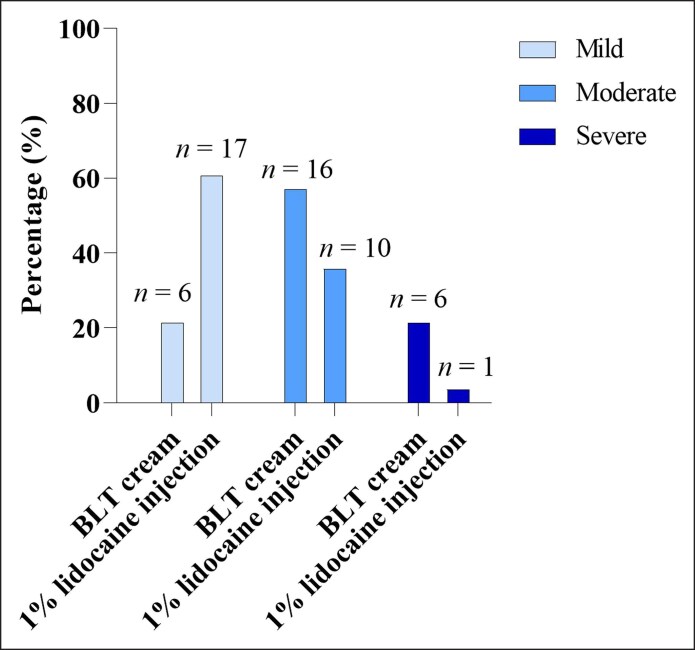
Distribution of pain severity levels—mild, moderate, and severe—following BLT cream and 1% lidocaine injection during facial aesthetic procedures, *n* = 28. The percentages of patients reporting each severity level are presented, with sample sizes (*n*) indicated above each bar. The 1% lidocaine injection was associated predominantly with mild pain, whereas BLT cream showed higher proportions of moderate and severe pain reports.

**Figure 2.**
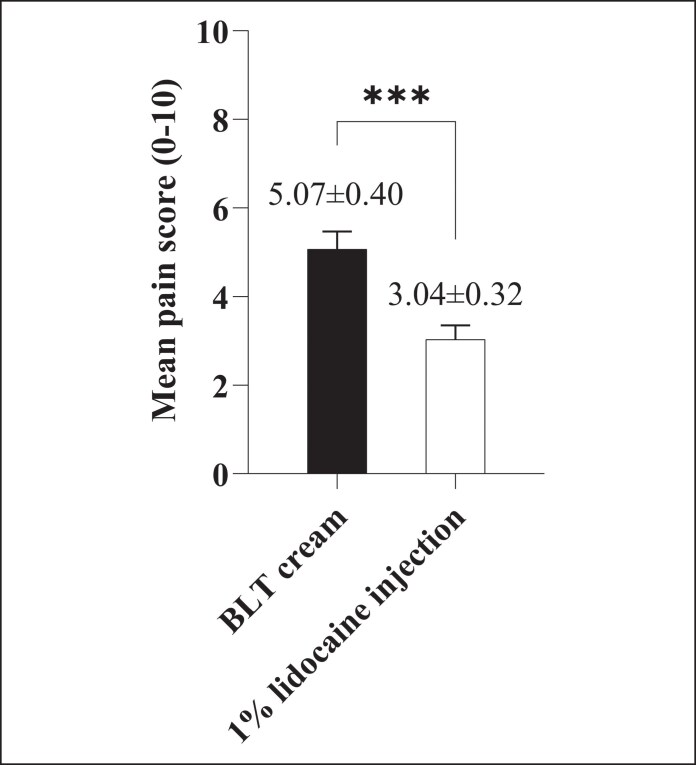
Mean pain scores during cannula-based facial treatment following BLT cream or 1% lidocaine injection, *n* = 28. Pain scores were significantly lower with lidocaine injection compared with BLT cream (*P* = .0001). Data are presented as mean ± standard error of mean. ***P < .001.

As shown in Table 2 and Figure 1, more than half of the patients reported lower pain levels with 1% lidocaine injection compared with BLT cream. Specifically, 16 patients (57.1%) reported moderate pain after application of BLT cream, whereas only 10 patients (35.7%) did so following 1% lidocaine injection. Additionally, severe pain was reported by 6 patients (21.4%) after BLT cream, compared with only 1 patient (3.6%) after lidocaine. Conversely, mild pain was more frequently reported following 1% lidocaine injection (*n* = 17, 60.7%) than after BLT cream (*n* = 6, 21.4%). The mean pain score was significantly higher with BLT cream (5.07 ± 0.40; 95% CI, 4.24-5.90) than with 1% lidocaine injection (3.04 ± 0.32; 95% CI, 2.38-3.69), with a mean difference of 2.03, *P* = .0001 (Figure 2).

**Table 2.** Distribution of Patients by Pain Severity for BLT Cream and 1% Lidocaine Injection Based on the Numerical Pain Rating Scale

Pain severity	BLT cream, *n* (%)	1% lidocaine injection, *n* (%)
None	0 (0.0)	0 (0.0)
Mild	6 (21.4)	17 (60.7)
Moderate	16 (57.1)	10 (35.7)
Severe	6 (21.4)	1 (3.6)

*n* = 28.

**Figure 3.**
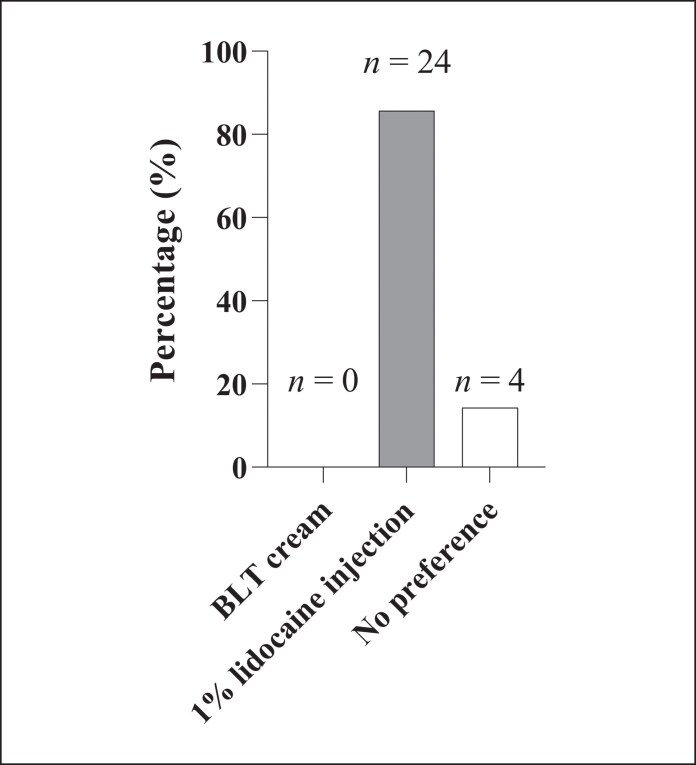
Patient preference for anesthetic methods during cannula-based facial treatment, *n* = 28. The majority of patients (*n* = 24, 85.7%) preferred 1% lidocaine injection, whereas a minority (*n* = 4, 14.3%) reported no discernible preference between the 2 methods.

Overall, the majority of patients (*n* = 24, 85.7%) expressed a preference for local anesthesia with 1% lidocaine injection, whereas none (*n* = 0, 0%) preferred BLT cream (Figure 3). Four patients (14.3%) reported no perceived difference between the 2 methods.

Both BLT cream and 1% lidocaine were well tolerated and demonstrated a favorable safety profile in this study. No major complications or adverse events were observed. A qualitative reduction in facial bruising was observed in 2 patients (7.1%) who received a 1% lidocaine injection compared with those treated with BLT cream following cannula-based facial treatments. No bruising or other significant local reactions were reported by the remaining patients.

## DISCUSSION

Dermal filler and skin booster injections are common cosmetic procedures sought by patients aiming to achieve a more youthful and attractive appearance. Although these treatments are minimally invasive, pain experienced during the procedure remains a frequent concern and can significantly impact the overall patient experience. A recent study by Shi and co-workers found that 42 (67.7%) physicians observed over half of their patients expressing concerns about injection pain, and 101 (82.1%) patients anticipated a pain score of 5 or higher before treatment.^24^ Therefore, effective pain management is essential, not only to reduce patient anxiety and fear but also enhance overall satisfaction with the procedure.^25^

In this retrospective study, we compared the anesthetic efficacy of BLT cream, a compounded topical anesthetic, with 1% lidocaine local injection in reducing procedural pain during cannula-based facial treatments; 1% lidocaine is among the most widely used local anesthetics because of its rapid onset (typically 1-3 min) and excellent safety profile. Discomfort associated with its injection can be minimized by using fine-gauge needles (27G or 30G) and slow administration.^26,27^ BLT cream combines 20% benzocaine, 10% lidocaine, and 4% tetracaine and is typically compounded by specialty pharmacies or in-house.^28^ Benzocaine offers rapid onset (within 30 s at a 20% concentration) but has a relatively short duration of effect (5-15 min). The addition of lidocaine and tetracaine helps to extend the overall anesthetic duration but exhibits a slower onset, averaging 10 to 15 min.^29,30^ Although previous studies have demonstrated that topical anesthetics can offer efficacy comparable to injectable anesthesia, making them the preferred choice among patients, our findings suggest otherwise.^28,31,32^ In our cohort, subcutaneous injection of 1% lidocaine provided significantly better pain relief compared with BLT cream, as evidenced by lower mean pain scores reported by patients. This suggests that lidocaine offers superior anesthetic efficacy for cannula-based facial treatments. These findings align with a previous study by Zdybski and Grodzka, who demonstrated that although topical application of BLT cream is noninvasive and easy to administer, it provides only a moderate anesthetic effect during dermocosmetic procedures, even when combined with premedication with 400 mg of ibuprofen.^33^

Furthermore, the difference in pain relief observed in our study was reflected in patient preferences, with most patients favoring 1% lidocaine injection over BLT cream for facial filler procedures. This preference reinforces the effectiveness of lidocaine in providing more consistent and sustained pain relief during such treatments. Additionally, the immediate onset of action with lidocaine injections, eliminating the waiting period needed for topical agents to take effect, also contributed to its favorability in the present study. Interestingly, our findings contrast with a recent study comparing 2% lidocaine infiltration with EMLA cream (lidocaine 2.5% and prilocaine 2.5%) before spinal needle insertion in pregnant women. Hameed and Khan reported that both the mean visual analog scale pain score, and the objective pain score were significantly lower in the EMLA group compared with the 2% lidocaine group, with a substantially higher proportion of patients in the EMLA group expressing greater overall satisfaction (76.7% vs 20.0%).^34^ This discrepancy may be attributed to differences in procedural context, types of topical anesthetic used, and patient populations. Notably, physiological changes during pregnancy might have influenced pain perception, potentially contributing to the differing outcomes.

It is also important to highlight that individual variations in anesthetic absorption rates play a critical role in determining anesthetic effectiveness. Anesthetic agents exert their effect primarily by inhibiting voltage-gated sodium channels within cutaneous nerve fibers, thereby blocking nerve depolarization and the subsequent pain signal transmission. These nerve fibers, located within the epidermis and dermis, are protected by the stratum corneum, a highly lipophilic, water-impermeable barrier that regulates the penetration and absorption of exogenous substances.^35^ Although topical anesthetic agents can readily penetrate the skin to provide local anesthesia, their absorption is influenced by several factors, including the acid dissociation constant (p*K*_a_) of the agent, application area, epidermal and dermal thickness, and the integrity of the skin barrier.^35-37^ Under normal physiological conditions, such as normal, healthy skin, only the uncharged (nonionized) form of the anesthetic can effectively diffuse through the lipophilic stratum corneum. Penetration is also more efficient in areas where the stratum corneum is thinner, such as the eyelids.^38^ Conversely, in inflamed tissues, the local pH decreases, shifting the equilibrium toward the ionized form, reducing the proportion of the nonionized form of the drug available to cross the stratum corneum and bind to the voltage-gated sodium channels, thereby diminishing the efficacy of anesthetic.^16,35^

Furthermore, unlike injected anesthetics, which bypass the stratum corneum lipid bilayers and directly reach the dermis for rapid onset, topical agents require more time to penetrate the skin and reach their target site. Notably, it has been reported that the transdermal absorption of the active compound in topical anesthetic formulations is limited, with only 10% to 20% permeating the skin, highlighting their reduced efficacy in deep tissue anesthesia.^39^ Although increasing the surface area of application may improve absorption efficacy and prolong anesthetic effect, excessive or prolonged application, especially at high concentrations, can increase the risk of adverse reactions and systemic toxicity.^35^ In 2006, the United States FDA warned that combining multiple anesthetics into a single product can amplify their harmful effects, with exposure to high concentrations of compounded creams linked to serious complications such as seizures and cardiac arrhythmias.^40^ Corneal damage has also been reported in a 64-year-old Caucasian woman following full-face application of BLT cream (20% benzocaine, 8% lidocaine, and 4% tetracaine) before skin resurfacing treatment.^41^ Among these agents, benzocaine has been shown to be associated with the highest incidence of side effects. One study reported allergic reactions in 26.6% of patients, erythema in 23.3%, and pruritus in 20%, rates significantly higher than those associated with lidocaine and tetracaine.^27^ These findings underscore the significant health risks associated with their improper use. Nevertheless, despite their widespread use, there are currently no standardized guidelines for the optimal dosing, formulation, or safety of compounded BLT creams. In fact, many available formulations exceed the concentrations considered safe and effective by the FDA.^42^ In contrast, local injection allows precise dosing, reduces the risk of over- or under-dosing, and avoids excessive systemic concentrations, thereby enhancing both the efficacy and safety of anesthesia.^43^ In our study, the small doses of BLT cream and 1% lidocaine injection used were well tolerated, with no major adverse events observed, highlighting the low clinical risk of the protocols employed.

Beyond safety and efficacy considerations, cost-effectiveness is an important factor influencing the choice of anesthetic method. Currently, a 100 g tub of compounded BLT cream costs approximately SGD 90 (≈USD 69), with each application requiring ∼3 g, allowing treatment of ∼33 patients at a cost of approximately SGD 2.70 (≈USD 2.07) per use. In contrast, a 10 mL bottle of 1% lidocaine costs around SGD 2.60 (≈USD 1.99) and can be used for up to 12 patients, resulting in a significantly lower cost of only SGD 0.22 (≈USD 0.17) per injection. This substantial cost further supports the use of 1% lidocaine injections as a more economical option for procedural anesthesia, without compromising its efficacy or safety.

Several limitations should be acknowledged in this study. First, the retrospective design may introduce recall bias, as pain assessments were collected based on patients’ recollection of their overall procedural experience rather than real-time reporting. Although reflective of common clinical documentation, this approach may reduce the accuracy of pain differentiation between procedural steps (eg, lidocaine injection, cannula insertion, and product delivery). A prospective study design with standardized pain scoring at multiple time points would provide a more objective evaluation of each component of the procedure. Second, the relatively small sample size (28 participants) and the inclusion of only female participants may limit the generalizability of the findings across genders and broader clinical settings. The modest sample size also increases the risk of the study being underpowered to detect subtle differences between procedural steps. Likewise, the single-center design further restricts applicability to broader patient populations. Third, methodological factors may have influenced pain outcomes. The study compared a 1% lidocaine injection with a 10 min application of BLT cream. Although this duration reflects common practice in many aesthetic clinics within our region, it may be shorter than the recommended 15 to 20 min used in other settings, which could potentially influence the degree of topical anesthesia achieved. Future studies exploring different application durations may help clarify the optimal protocol for topical anesthetic use in aesthetic procedures. Additionally, BLT cream was always applied first, followed by a 1% lidocaine injection, which may have introduced an order effect, with patients’ previous exposure to the procedure could have influenced their perception of pain during the second treatment, potentially lowering reported pain scores. Pain scores reflected the patient's overall experience, including discomfort from lidocaine injection, needle entry, and cannula movement. Although this provides a realistic measure of patient-perceived comfort during the entire session, it may not allow precise isolation of the pain contributed by each procedural component. Similarly, bruising was assessed observationally, which may introduce subjectivity and bias in reporting. Future studies could improve accuracy by incorporating standardized photographic documentation, multispectral imaging combined with observer-based scoring, or validated scoring tools such as the Bruise Visibility Scale.^44,45^ Finally, pain perception is inherently subjective and influenced by multiple factors, such as psychological state, anxiety, age, skin thickness, and subcutaneous fat distribution. These variables may affect perceived pain levels and response to anesthetic techniques and, when combined with the small and relatively homogenous sample, may introduce additional variability that limits the external validity of the findings.^8,46^

Future prospective, randomized studies with larger and more diverse populations, real-time pain assessments, and controlled topical anesthetic durations are warranted to confirm these findings and further validate the safety and efficacy of both anesthetic approaches. To improve the accuracy of pain evaluation, future studies should consider incorporating physiological markers, such as blood pressure, heart rate, respiratory rate, oxygen saturation, and pupil diameter, alongside self-reported pain scores. These markers could provide a more reliable assessment of anesthetic effectiveness and help address the limitations associated with subjective reporting.^47^ Lastly, the potential impact of anesthetic agents on the efficacy of injectable compounds was not assessed in this study. Although some studies have demonstrated that the addition of local anesthetics to filler injections does not affect their pharmacokinetic characteristics or overall efficacy, the topical application of betacaine before botulinum toxin type A (BTX-A) injection has been reported to not only provide inadequate pain relief but also appear to reduce the therapeutic efficacy of BTX-A.^48-50^ To the best of our knowledge, the effect of local anesthesia before filler injection has not yet been determined. Future studies should explore this interaction further to assess whether the injection of anesthetic agents influences the long-term outcomes or effectiveness of injectable compounds.

## CONCLUSIONS

In conclusion, this preliminary report demonstrates that local injection of 1% lidocaine provides significantly superior anesthesia and greater patient preference compared with the use of compounded BLT cream during cannula-based facial treatment. Further studies with larger sample sizes and diverse populations are warranted to validate these preliminary findings. Such studies will be instrumental in guiding physicians in selecting the most effective and safe local anesthetic agents for clinical use.
